# Neuroimaging features of whole‐brain functional connectivity predict attack frequency of migraine

**DOI:** 10.1002/hbm.24854

**Published:** 2019-11-04

**Authors:** Junya Mu, Tao Chen, Shilan Quan, Chen Wang, Ling Zhao, Jixin Liu

**Affiliations:** ^1^ Center for Brain Imaging, School of Life Science and Technology Xidian University Xi'an China; ^2^ Engineering Research Center of Molecular and Neuro Imaging Ministry of Education Xi'an China; ^3^ Acupuncture and Tuina School Chengdu University of Traditional Chinese Medicine Chengdu Sichuan China

**Keywords:** attack frequency, functional connectivity, logistic regression, migraine, prediction

## Abstract

Migraine is a chronic neurological disorder characterized by attacks of moderate or severe headache accompanying functionally and structurally maladaptive changes in brain. As the headache days/month is often measured by patient self‐report and tends to be overestimated than actually experienced, the possibility of using neuroimaging data to predict migraine attack frequency is of great interest. To identify neuroimaging features that could objectively evaluate patients' headache days, a total of 179 migraineurs were recruited from two data center with one dataset used as the training/test cohort and the other used as the validating cohort. The guidelines for controlled trials of prophylactic treatment of chronic migraine in adults were used to identify the frequency of attacks and migraineurs were divided into low (MOl) and high (MOh) subgroups. Whole‐brain functional connectivity was used to build multivariate logistic regression models with model iteration optimization to identify MOl and MOh. The best model accurately discriminated MOh from MOl with AUC of 0.91 (95%CI [0.86, 0.95]) in the training/test cohort and 0.79 in the validating cohort. The discriminative features were mainly located within the limbic lobe, frontal lobe, and temporal lobe. Permutation tests analysis demonstrated that the classification performance of these features was significantly better than chance. Furthermore, the indicator of functional connectivity had a higher odds ratio than behavioral variables with implementing a holistic regression analysis. The current findings suggested that the migraine attack frequency could be distinguished by using machine‐learning algorithms, and highlighted the role of brain functional connectivity in revealing underlying migraine‐related neurobiology.

## 1 INTRODUCTION

Migraine is a neurological disorder characterized by attacks of moderate or severe headache, and can be divided into episodic migraine (attacks that occur ≤15 days/month) and chronic migraine (attacks that occur ˃15 days/month) (Dodick, 2018). Without effective treatments, patients with episodic migraine would experience more headache attacks and even develop chronic migraine (Lipton et al., 2015; Lipton & Silberstein, 2015). Since attack frequency is a risk factor for headache progression, it is possible that preventive medication may reduce the risk of progression to chronic migraine (Dodick, 2018; Lipton et al., 2007). The headache days per month is often evaluated in the clinical contexts by patient self‐report, which may rely on patient memory (Houle et al., 2013; Niere & Jerak, 2004). However, memories may be enhanced to various extents, causing the measurement of headache days to become unreliable or inaccurate (i.e., recall headache days may be higher than the actual headache days) (Berger et al., 2018; Haywood et al., 2018). An objective measurement of headache days per month might be helpful in the decision‐making process of providing preventive medication for migraine patients.

Migraine is mainly a disorder of brain function that involves the brain regions of sensory discrimination of pain, affective‐emotional processing, cognitive processing, and pain modulation (Schwedt, Chiang, Chong, & Dodick, 2015), which serve as an integrated network in its development and maintenance (Liu et al., 2017). Functional magnetic resonance imaging (fMRI) can be used to non‐invasively acquire the data of brain activities, which would be useful to explore the central mechanisms underlying migraine (Fox & Raichle, 2007). Synchronous fluctuations in the blood oxygen‐level‐dependent (BOLD) signal measured by fMRI are deemed to reflect functional connectivity, or functional communication between brain regions (Rosenberg et al., 2016; Schwedt et al., 2015). Compared with the analysis using only several predefined regions or networks of interest, whole‐brain functional connectivity analysis can ensure the optimal use of the pattern information of the brain (Zeng et al., 2012). Neuroimaging studies have consistently shown the abnormal brain functional network organization in patients with migraine (Liu et al., 2015; Maleki et al., 2012; Schwedt et al., 2015; Yuan et al., 2013), and these connection patterns may sharp the cognition and behavior to individual differences in migraine vulnerability (Kucyi & Davis, 2015). Hence, it is possible to locate the neuroimaging features in the brain functional network, which could be used to objectively evaluate the headache days per month (Fox & Raichle, 2007). Furthermore, the machine‐learning‐based predictive model does not require months of journaling to determine prognosis, and could help predict headache days for clinical settings without the presence of patient‐reported ratings.

In the current study, to identify neuroimaging features that could objectively evaluate patients' attack frequency of migraine, we built a multivariate logistic regression model by using the brain functional connectivity data, in which a total of 179 migraineurs without aura (MO) were recruited from two data centers with one dataset used as the training/test cohort (*N* = 151) and the other used as the validating cohort (*N* = 28).

## 2 MATERIALS AND METHODS

All research procedures were approved by the West China Hospital Subcommittee on Human Studies and the Medical Ethics Committee of the Affiliated Hospital of Chengdu University of Traditional Chinese Medicine and were conducted in accordance with the Declaration of Helsinki. All participants gave written, informed consent after the experimental procedures had been fully explained.

### 2.1 Participants

Inclusion criteria for the migraine patients were according to the International Classification of Headache Disorders third edition (beta version) (2013): (a) migraine attacks last 4–72 hours (untreated or unsuccessfully treated); (b) featuring at least two of the following characteristics: unilateral location, pulsating quality, moderate to severe pain intensity and aggravation by causing avoidance of routine physical activity; and (c) there is nausea and/or vomiting, or photophobia and phonophobia during migraine. Exclusion criteria were: (a) any physical illness such as a brain tumor, hepatitis, or epilepsy as assessed according to clinical evaluations and medical records; (b) existence of other comorbid chronic pain conditions (e.g., tension type headache, fibromyalgia, etc.); (c) existence of a neurological disease or psychiatric disorder; (d) pregnancy; (e) use of prescription medications within the last month; (f) alcohol, nicotine, or drug abuse; and (g) claustrophobia.

A total of 151 MO patients (age: 28.69 ± 0.83 years, 39 males and 112 females) were recruited from the West China Hospital, and these patients were used as the training/test cohort. Another 28 MO patients (age: 31.43 ± 1.72 years, 8 males and 20 females) were recruited from the Chengdu University of Traditional Chinese Medicine and the surrounding community, and were used as the validating cohort. All patients were right‐handed and were evaluated by a neurologist. Patients were instructed to complete a daily headache diary every evening before retiring. The diaries were returned to the researchers at the end of a 4‐week period. Telephone reminders were given if diaries were not received within 2 days of the end of the four‐week period. During the 4 weeks before the MRI scans, patients carefully rated the average headache intensity (0–10 scale, 10 being the most intense pain imaginable), headache days and migraine attack duration (hours) with the migraine diary. The Zung Self‐Rating Anxiety Scale (SAS) and Zung Self‐Rating Depression Scale (SDS) were used to quantify anxiety/depression‐related symptoms of the patients. Analysis of migraine diaries was performed by two blinded evaluators.

### 2.2 Imaging acquisition

For the training/test cohort, MRI data acquisition was carried out in a 3.0 Tesla Signa GE scanner with an 8‐channel phase head coil at the Huaxi MR Research Center, Chengdu, China. The resting‐state functional images were obtained with echo‐planar imaging (EPI) (30 continuous slices with a slice thickness = 5 mm, TR = 2000 ms, TE = 30 ms, flip angle = 90°, FOV = 240 mm × 240 mm, matrix = 64 × 64). In addition to functional imaging, a high‐resolution T1 scan was acquired for anatomic normalization. T1 structural image for each subject was acquired using an axial Fast Spoiled Gradient Recalled sequence (TR = 1900 ms, TE = 2.26 ms, data matrix = 256 × 256, FOV = 256 mm × 256 mm, voxel size = 1 × 1 × 1 mm^3^).

For the validating cohort, imaging acquisition was carried out in a 3.0 Tesla Siemens magnetic resonance scanner with an 8‐channel phase array head coil at the Huaxi MR Research Center. Resting‐state functional images were obtained with a gradient echo EPI sequence with the following parameters: TR = 2000 ms; TE = 30 ms; flip angle = 90°; 30 continuous slices with a slice thickness = 5 mm; data matrix = 64 × 64; FOV = 240 mm × 240 mm. For each subject, a total of 205 volumes were acquired, resulting in a total scan time of 410 s. A high resolution T1 structural image for each participant was acquired by using a three‐dimensional MRI sequence with a voxel size of 1 × 1 × 1 mm^3^ employing an axial Fast Spoiled Gradient Recalled sequence with the following parameters: TR = 1,900 ms; TE = 2.26 ms; data matrix = 256 × 256; FOV = 256 mm × 256 mm.

### 2.3 fMRI data preprocessing

Functional data were preprocessed by the Statistical Parametric Mapping package (SPM12, https://www.fil.ion.ucl.ac.uk/spm/software/spm12). The first five volumes were discarded to eliminate nonequilibrium effects of magnetization and to allow participants to become familiar with the scanning circumstances. Functional volumes were then slice time‐corrected and realigned, and normalized to the Montreal Neurological Institute template brain, and smoothed with a 6‐mm^3^ isotropic Gaussian kernel. Individuals with an estimated maximum displacement in any direction larger than 1 mm or head rotation larger than 1° were discarded from the study, and no data were excluded under this criterion. A band‐pass filter (0.01 Hz < f < 0.1 Hz) was applied to remove the effects of low‐frequency drift and high frequency physiological noise. Finally, regression of nuisance covariates including Friston 24 head movement parameters, cerebrospinal fluid signals and white matter signals from the fMRI data were carried out. The data used to support the findings of this study are available from the corresponding author upon request.

### 2.4 Head‐motion calculations

To test whether our observations would be held when considering the effects of head motion, we eliminated volumes from each subject's resting fMRI time series that were associated with sudden head motion. An index of framewise displacement (FD) was applied to mark the volumes that tended to behave as burst noise. This would result in temporal masks for our data and similar approaches have been used in several previous rs‐fMRI studies (Liu et al., 2015; Power, Barnes, Snyder, Schlaggar, & Petersen, 2012). For each subject, the flag volume was censored if its derivative values were above 0.5 (Power et al., 2012).

### 2.5 Functional connectivity measures

The Human Brainnetome Atlas (Fan et al., 2016) containing 210 cortical and 36 subcortical regions of interest (ROIs) was used to create fMRI time course correlation matrices (i.e., functional connectivity matrices) for the participants' processed echo‐planar image time series. Time series were extracted and averaged within each region. For each participant, Pearson correlation coefficients were calculated between the average time series of each region in the atlas to form a 246 × 246 symmetrical matrix (30,135 unique connections). All correlation values were converted using a Fisher Z‐transformation to remove the effects of global levels of correlation (Plitt, Barnes, Wallace, Kenworthy, & Martin, 2015). The GRETNA toolbox (https://www.nitrc.org/projects/gretna), implemented in Matlab (MathWorks, Inc.), was used for the construction of functional connectivity matrices (Wang et al., 2015). The 30,135 unique connections for each participant were used as the initial features. Considering the effects of age and sex, a linear regression model was applied to obtain an age‐ and sex‐adjusted feature for each connection.

### 2.6 Grouping threshold definition

According to guidelines for controlled trials of prophylactic treatment of chronic migraine in adults, preventive medications should be given when migraine attacks are frequent (Dodick, 2018; Silberstein et al., 2008). Specifically, the migraineur who had greater than or equal to 8 headache days per month should take preventive medications, which may reduce the risk of progression to chronic migraine (Dodick, 2018; Silberstein et al., 2008). Thus, the frequency of 8 headache days/month was used as the grouping threshold in our study.

### 2.7 Multivariate logistic regression model

**Figure 1:**
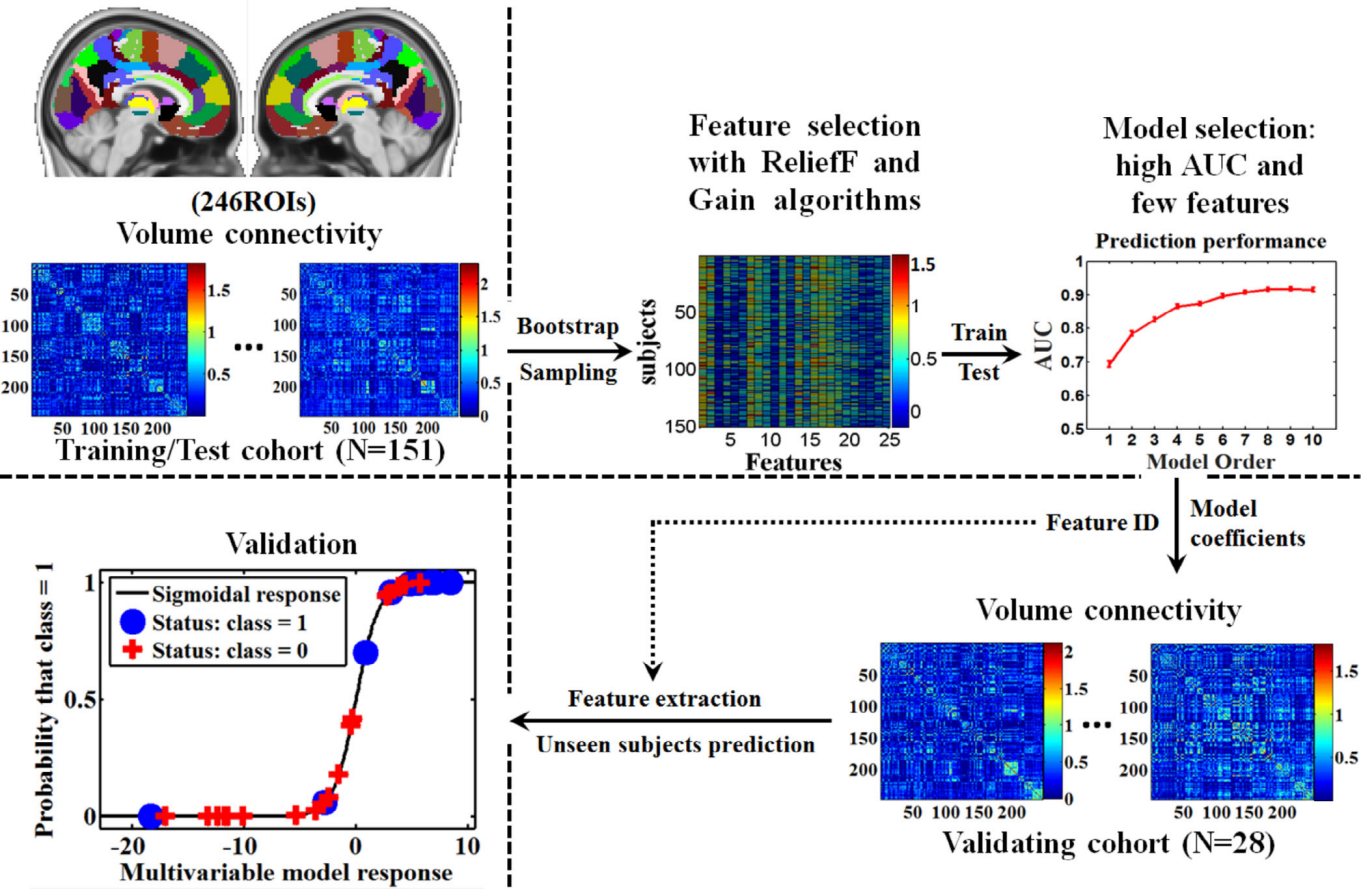
Schematic overview of the multivariate logistic regression analysis. Whole‐brain functional connectivity matrix was computed for each subject using a 246‐region cortical and subcortical atlas, for a total of 30,135 unique features. The training/test cohort (*N* = 151) was used to build a multivariate logistic regression model with bootstrap sampling and ReliefF and Gain algorithms‐based feature selection procedures. The area under the receiver operating characteristic curve (AUC) was used to select the best model that had a high AUC and few features. Then, the same features were extracted for the validating cohort (*N* = 28) according to the feature index of the best model. Finally, the group label for the unseen subjects was calculated using the regression coefficients of the best model

Following the general methodology developed by Vallières et al (Vallières, Freeman, Skamene, & El Naqa, 2015), an adapted method was applied to find the migraine attack frequency‐related neuroimaging features. The schematic overview can be seen in Figure 1. Specifically, the training/test cohort was divided into MOl (MO with a low attack frequency, that is, headache days < 8 per month) group and MOh (MO with a high attack frequency, that is, headache days ≥ 8 per month) group (Table 1). Multivariate models were built for the initial features of the training/test cohort and modeled outcome using imbalanced‐adjusted logistic regression. The initial feature set first underwent feature dimension reduction using a ReliefF algorithm (Robnik‐Šikonja & Kononenko, 2003), which reduced the feature dimension from 30,135 to 10,000. For short, every feature was assigned with a weight value indicating its relevance to the group label, and the first 10,000 features were remained. Then, Gain algorithm‐based feature selection was performed using 100 bootstrap training samples to yield a reduced feature set of 25 outcome‐related but low‐redundancy features (Vallières et al., 2015). After feature selection, stepwise forward model construction was conducted by maximizing the 0.632+ bootstrap area under the receiver operating characteristic curve (AUC) metric in 1000 bootstrap training and testing samples (training sample: test sample≈2:1) to obtain logistic regression models combining 1 to 10 features (corresponding to model order: 1 to 10) (Vallières et al., 2015). For the outcome, the model providing the combination of functional connection features with the best parsimonious properties (i.e., the minimum model order and the maximum AUC) was chosen. Finally, the classification performance of the chosen model (i.e., best model) was estimated using the average AUC, sensitivity, specificity, and accuracy obtained in 1000 bootstrap testing samples.

**Table 1:** Demographic and headache information for training/test cohort with an imbalanced grouping

	MOl (*N* = 102)	MOh (*N* = 49)	*p*
Age (years)	28.04 ± 0.99	30.04 ± 1.51	.26^a^
Sex (M/F)	23/79	16/33	.18^b^
Education (years)	15.13 ± 0.20	15.36 ± 0.29	.89^a^
Disease duration (monthes)	96.19 ± 7.40	97.51 ± 10.13	.92^a^
Migraine attacks during past 4 weeks			
Headache days	3.37 ± 0.15	10.45 ± 0.40	.00^a^
Average duration of a migraine attack (hours)	9.71 ± 1.10	11.12 ± 1.36	.45^a^
Average pain intensity (0–10)	5.43 ± 0.17	5.63 ± 0.23	.48^a^
SAS	45.06 ± 0.88	48.58 ± 1.55	.04^a^
SDS	43.33 ± 1.07	47.42 ± 1.60	.03^a^

*Note*: Data are presented as mean ± *SE*.

Abbreviations: MOl, migraineurs without aura with a lower attack frequency (i.e., headache days **<** 8); MOh, migraineurs without aura with a higher attack frequency (i.e., headache days ≥8); SAS, self‐rating anxiety scale; SDS, self‐rating depression scale.

a *p*‐value established through a two sample *t*‐test.

b *p*‐value established through a chi‐square test.

To further evaluate the generalizability of the best model, we used a validating cohort (28 MO, containing 18 MOl (headache days <8) and 10 MOh (headache days ≥ 8)). The same functional connections were extracted from the validating cohort, and then fed into the logistic regression model. The AUC, sensitivity, specificity, and accuracy were computed.

### 2.8 Robustness of the features

To test whether the classification performance of the true features of the best model was higher than chance level and whether counterpart random features of functional connectivity could distinguish MOl from MOh, the random connectivity matrices with the same degree distribution as the real network were generated. Specifically, the feature matrix of the training/test cohort was extracted from the best model, and the corresponding label vector (i.e., 0 for the MOl group and 1 for the MOh group) was randomly shuffled. Then, the corresponding training and test sets of the best model were used to train and test the model, respectively. As for the random features, the same dimension random connectivity matrix (i.e., 246 × 246 matrix) for each participant was first generated by using the GRETNA toolbox to ensure the same degree distribution as the real network; the random features that were located within the same location of the connection matrix were extracted to conduct permutation tests. Then, the coefficients of the logistic regression model were computed using the shuffled training sample, and the classification performance was tested on the shuffled test sample. The null distribution of the classification AUC for the training/test cohort was built.

### 2.9 Additional validation

To evaluate the influence of an imbalanced grouping, we adjusted the grouping threshold to 5 headache days/month (the median of headache days) of the training/test cohort to insure a balanced grouping. Specifically, the training/test cohort was divided into a MOl (MO with a lower attack frequency, that is, headache days < 5) group and a MOh (MO with a higher attack frequency, that is, headache days ≥ 5) group (Table S1). Then, the feature selection, model selection, and model generalizability evaluation procedures described in the *Multivariate logistic regression model* part were repeated. Note that the grouping threshold of headache days was also adjusted to 5 for the validating cohort (i.e., 28 MO, containing 15 MOl (headache days <5) and 13 MOh (headache days ≥ 5)).

### 2.10 Development of a holistic evaluation model

To explore the relationship between migraine attack frequency and demographical, behavioral and neuroimaging indicators, an individual logistic regression analysis was performed. According the best classification model, a neuroimaging score was calculated for each participant via a linear combination of selected functional connectivity features weighting by its corresponding regression coefficients (Huang et al., 2016). Then, an individual logistic regression analysis was conducted with the following candidate indicators: age, sex, disease duration, average duration of a migraine attack, average pain intensity, SAS, SDS, and neuroimaging score. A backward stepwise selection was adopted and the likelihood ratio test with Akaike's information criterion was used to determine the stopping rule (Huang et al., 2016).

### 2.11 Statistical analysis

As for the demographic and headache information, group comparison was carried out using a two‐sample *t*‐test and chi‐square test in SPSS 20.0 (SPSS Inc., Chicago, Illinois) with a statistical power *p* < .05.

As for permutation tests, after 5,000 permutations, the significance value *p* for each metric (i.e., AUC, sensitivity, specificity, and accuracy) was computed by dividing the number of times that showed a higher value than the actual value derived from the nonpermuted model by the total number of permutations, and *p* < .05 was accepted(Plitt et al., 2015).

## 3 RESULTS

### 3.1 Demographic and headache information

A total of 179 MO (i.e., the training/test cohort (*N* = 151) and the validating cohort (*N* = 28)) were enrolled in this study. For the training/test cohort (both imbalanced and balanced grouping), there were no significant differences between the MOl and MOh groups with regard to age, sex, education, disease duration, average duration of a migraine attack, and average pain intensity (*p* > .05, Table 1 and S1). For the validating cohort, the age was 31.43 ± 1.72 years (mean ± *SE*), and the headache days during past month were 7.82 ± 1.25 days (mean ± *SE*) (Table 2).

**Table 2:** Demographic and headache information for validating cohort

	MO (*N* = 28)
Age (years)	31.43 ± 1.72
Sex (M/F)	8/20
Education (years)	14.93 ± 0.30
Disease duration (monthes)	99.57 ± 14.86
Migraine attacks during past 4 weeks	
Headache days	7.82 ± 1.25
Average duration of a migraine attack (hours)	19.30 ± 3.03
Average pain intensity (0–10)	5.59 ± 0.30
SAS	46.19 ± 1.53
SDS	45.64 ± 1.82

*Note*: Data are presented as mean ± *SE*.

Abbreviations: MO, migraineurs without aura; SAS, self‐rating anxiety scale; SDS, self‐rating depression scale.

### 3.2 Functional connectivity‐based classification of migraine attack frequency

**Figure 2:**
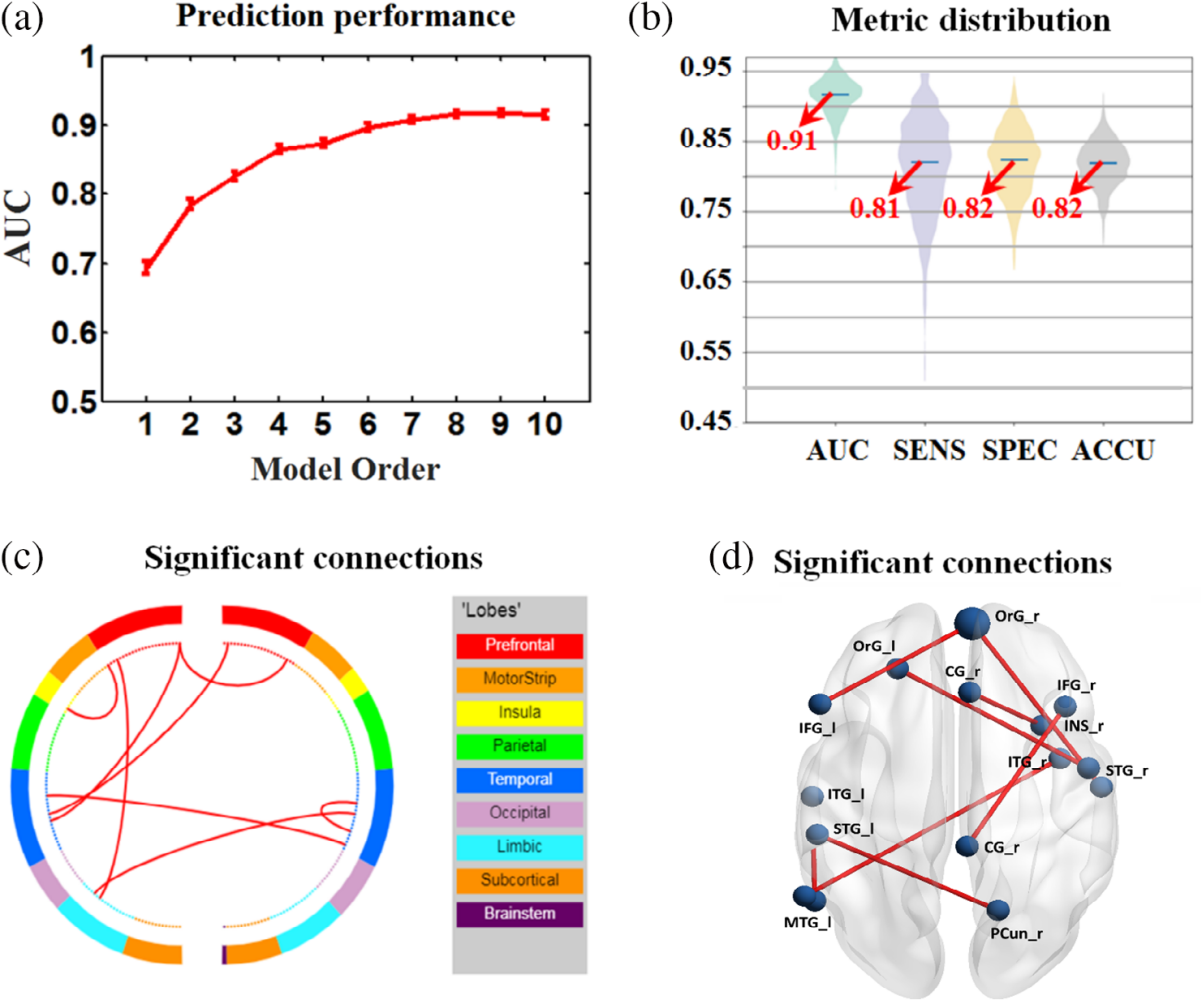
Model classification performance and migraine attack frequency‐related functional connections. (a) Estimation of classification performance is shown for combinations of 1 to 10 brain features (model orders) in terms of the AUC metric for the training/test cohort. (b) The metric distribution of the best model (i.e., model order 8) was shown with all mean values above 0.8. (c) Circle plots: nodes are color coded according to the cortical lobes, and the observed functional connections (edges) are drawn between the nodes. (d) Glass brain plots: each node is represented as a sphere, where the size of the sphere indicates the number of edges emanating from that node

The following results are for the training/test cohort using the imbalanced grouping (i.e., grouping threshold = 8 headache days/month), unless otherwise specified. No significant between group difference was found for the mean FD. Functional connectivity matrix for each participant was measured before the model construction. After the feature selection and model selection procedures, we found that the best model that could accurately discriminate MOh from MOl contained 8 features, which were mainly located within the limbic lobe, frontal lobe, and temporal lobe (Figure 2, Table 3). In the training/test cohort, the AUC, sensitivity, specificity, and accuracy were 0.91 (95% CI, 0.86 to 0.95), 81.29% (95% CI, 68.66% to 91.23%), 81.92% (95% CI, 73.76% to 88.96%), and 81.79% (95% CI, 76.01% to 86.77%), respectively (Table 4). As for the validating cohort, the AUC, sensitivity, specificity, and accuracy were 0.79, 80.00%, 72.22%, and 75.00%, respectively.

**Table 3:** Functional connections for migraine attack frequency prediction from the best model

Nodes of each pairwise functional connection				Model coefficient
Node name A	MNI coordinates (X,Y,Z)	Node name B	MNI coordinates (X,Y,Z)	
Cingulate gyrus (dorsal area 23)	(4, −37, 32)	Inferior frontal gyrus (opercular area 44)	(42, 22, 3)	−19.76
Orbital gyrus (medial area 11)	(6, 57, −16)	Inferior frontal gyrus (caudal area 45)	(−53, 23, 11)	11.09
Inferior temporal gyrus (ventrolateral area 37)	(−55, −60, −6)	Inferior temporal gyrus (intermediate lateral area 20)	(−56, −16, −28)	−11.27
Inferior temporal gyrus (rostral area 20)	(40, 0, −43)	Middle temporal gyrus (dorsolateral area 37)	(−59, −58, 4)	−9.83
Superior temporal gyrus (rostral area 22)	(56, −12, −5)	Orbital gyrus (medial area 11)	(6, 57, −16)	9.38
Superior temporal gyrus (TE1.0 and TE1.2)	(51, −4, −1)	Orbital Gyrus (lateral area 11)	(−23, 38, −18)	7.04
Cingulate gyrus (pregenual area 32)	(5, 28, 27)	Insular gyrus (ventral agranular insula)	(33, 14, −13)	−7.96
Precuneus (dorsomeidal parietooccipital sulcus)	(16, −64, 25)	Superior temporal gyrus (area 41/42)	(54, −24, 11)	−6.54

**Table 4:** Prediction performance of the best model

	Mean	95% CI
AUC	0.91	[0.86, 0.95]
Sensitivity	81.29%	[68.66%, 91.23%]
Specificity	81.92%	[73.76%, 88.96%]
Accuracy	81.79%	[76.01%, 86.77%]

*Note*: One thousand bootstrap samplings.

Abbreviations: AUC, the area under the receiver operating characteristic curve; CI, confidence interval.

**Figure 3:**
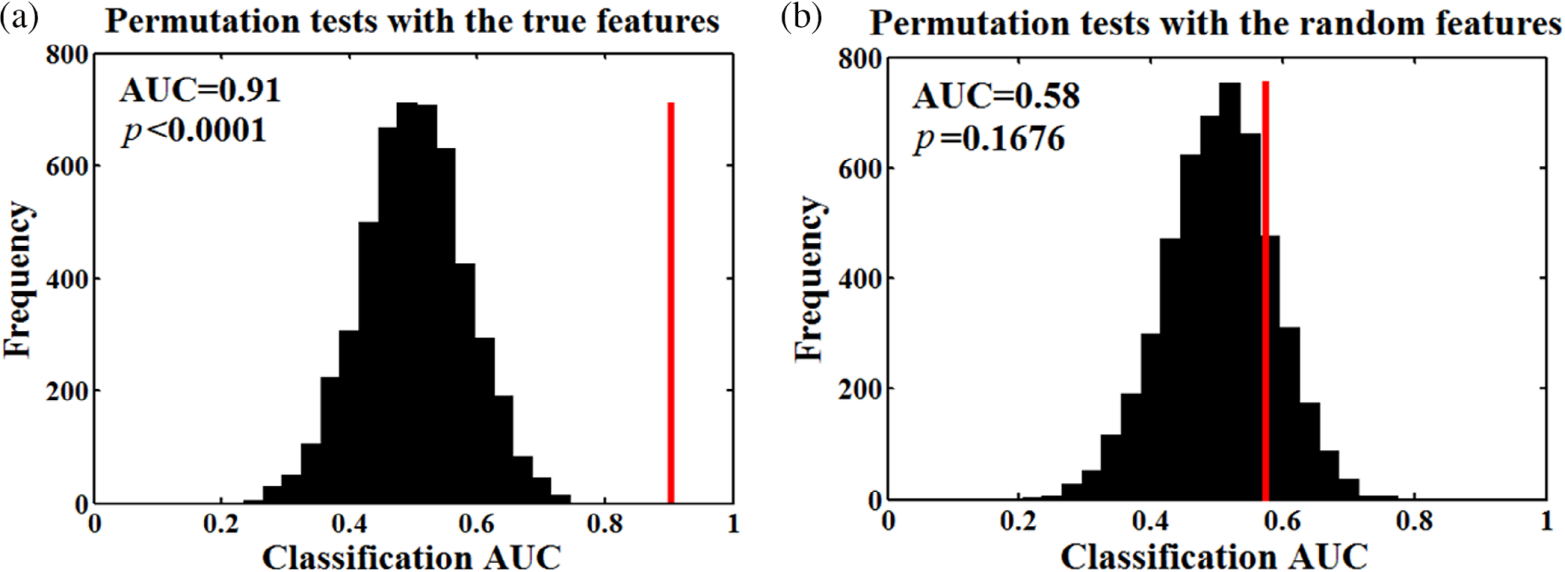
Classification AUC (indicated by a vertical red line) and corresponding null distribution with 5,000 random permutations. (a) The AUC was significantly greater than chance level (*p* < .0001) using the true features. (b) The AUC wasn't significantly greater than chance level (*p* = .1676) using the random features

To further evaluate the robustness of the discriminative features, we generated the counterpart random features of functional connectivity. The random connection matrix was generated for each participant with the guarantee of the same degree distribution to the real network. As for the true features, classification performance was significantly better than chance with the AUC, sensitivity, specificity and accuracy were 0.91, 83.34%, 82.22%, and 82.60%, respectively (*p* < .005, 5,000 permutations). However, as for the random features, the classification performance wasn't significantly better than chance; the AUC, sensitivity, specificity and accuracy were 0.58, 54.18%, 60.62%, and 58.54%, respectively (*p* > .05, 5,000 permutations). Additionally, the null distribution of the AUC was depicted with a histogram and the nonpermutation AUC was presented by a red line (Figure 3).

### 3.3 Classification with balanced grouping

Considering the influence of an imbalanced grouping, another grouping threshold of headache days was adopted to obtain a balanced grouping for the training/test cohort (i.e., 151 MO, containing 77 MOl (headache days < 5) and 74 MOh (headache days ≥ 5), Table S1). Through the same feature selection and model selection procedures, we found that the best model contained seven features. Compared with the model that trained with an imbalance‐adjusted strategy, this model obtained a relative moderate classification in the training/test cohort with the AUC, sensitivity, specificity, and accuracy being 0.81 (95% CI, 0.73 to 0.86), 73.58% (95% CI, 61.60% to 83.65%), 74.47% (95% CI, 62.67% to 83.93%), and 74.05% (95% CI, 67.47% to 79.80%), respectively (Table S2). As for the validating cohort, the AUC, sensitivity, specificity, and accuracy were 0.56, 100.00%, 0.00%, and 46.67%, respectively, which seemingly failed to distinguish MOh from MOl in the unseen dataset.

### 3.4 Holistic evaluation

We finally investigated the relationship between migraine attack frequency and demographical, behavioral and neuroimaging indicators from an aspect of holistic evaluation, and we found that the functional connectivity had a higher odds ratio than other variables (Table 5). Additionally, the *β* value corresponding to the neuroimaging score was positive (*β* = .666, *p* < .001, Table 5), indicating that the aberrant brain functional connectivity patterns were an influence factor for frequent migraine attacks.

**Table 5:** Logistic regression analysis with combined variables

Intercept and variable	*β*	Waldχ^2^	Odds ratio (95% CI)	*p*
Intercept	−1.138	0.399		.53
Age	0.022	0.297	1.022 (0.946 to 1.104)	.59
Sex	0.434	1.682	1.543 (0.801 to 2.974)	.20
Disease duration	−0.001	0.077	0.999 (0.988 to 1.009)	.78
Average duration of a migraine attack	0.038	3.100	1.038 (0.996 to 1.083)	.08
Average pain intensity	0.010	0.003	1.010 (0.692 to 1.474)	.96
SAS	−0.024	0.256	0.976 (0.889 to 1.072)	.61
SDS	0.019	0.179	1.019 (0.935 to 1.110)	.67
Neuroimaging score	0.666	27.191	1.947 (1.515 to 2.500)	<.001

Abbreviations: CI, confidence interval; SAS, self‐rating anxiety scale; SDS, self‐rating distress scale.

## 4 DISCUSSION

In this study, whole‐brain functional connectivity was used to build a multivariate logistic regression model to find migraine attack frequency (i.e., headache days per month) related neuroimaging features. Our model successfully predicted migraine attack frequency in independent datasets, demonstrating that the neuroimaging feature‐based model is generalizable for individual disease characterization in migraineurs. Furthermore, an individual logistic regression analysis using the combined variables (i.e., age, sex, disease duration, average duration of a migraine attack, average pain intensity, SAS, SDS, and neuroimaging score) indicated that the aberrant brain functional connectivity patterns were an influence factor for frequent migraine attacks.

### 4.1 Reliable identification of lower or higher frequency of migraine attacks

Preventive medications are of use to reduce the frequency and severity of attacks in people with frequent migraine (Dodick, 2018), and one of the criteria for considering or offering prevention was based on headache frequency (Lipton et al., 2007). Several studies characterize the extent of measurement error arising from rounding in headache frequency reporting (Houle et al., 2013), and pointed out that recall error in describing and quantifying pain was sufficiently common to distort the resulting population estimates in headache chronification research (Turner, Smitherman, Penzien, Lipton, & Houle, 2013). Although reliable and accurate measurement of pain could potentially improve treatment strategies and design more effective clinical trials, studies regarding predicting interindividual variability in migraine attack frequency are sparse. For better decision‐making in providing preventive medication for migraine patients, psychological and social factors such as anger, anger‐expression, anxiety, and depression have been used to predict the frequency of migraine attacks, but only obtained an accuracy of 69.8% (Bernardy et al., 2007). Due to a lack of sensitive predictive features, these studies using behavioral indicators to predict headache attacks seemingly failed to achieve good performance.

Borsook and colleagues proposed an allostatic model to understand the effect of high‐frequency headache attacks, and pointed out that repeated migraine attacks and physical or psychological stressors may cause progressive functional and structural changes in brain networks (Borsook, Maleki, Becerra, & McEwen, 2012). In our previous study, we investigated how the between‐group differences of functional connections in MO were organized along with the changing trend by using resting‐state fMRI, and found that the presence of chronic headache altered the functional connectivity from the local central nervous system due to a disruption in whole‐brain networks with increased disease duration (Liu et al., 2015). In the current study, we extended our previous findings and observed that the variability of headache days in migraineurs could be predicted from whole‐brain functional connectivity. The neuroimaging feature‐based multivariate logistic regression model obtained good performance in distinguishing MOl from MOh (AUC = 0.91 and 95% CI, 0.86 to 0.95). It should be noted that 81.29% of 49 MOh and 81.92% of 102 MOl were correctly classified, although the training/test cohort was imbalanced. We further showed that the same neuroimaging features could predict migraine attack frequency in an independent cohort of individuals with MO, which may demonstrate a potential of functional connectivity for identifying low and high migraine attack sufferers.

### 4.2 Consideration of the grouping threshold

According to the guidelines for controlled trials of prophylactic treatment of chronic migraine in adults (Dodick, 2018; Silberstein et al., 2008), a frequency of 8 headache days per month was used as the grouping threshold to subdivide our training/test cohort into a MOl group (102 participants) and MOh group (49 participants). For better learning from imbalanced data, prior probabilities for each group were set equally with an imbalance‐adjusted bootstrap approach (Vallières et al., 2015; Zhou et al., 2017). The results demonstrated that both the training/test cohort and validating cohort obtained good classification performance. Nevertheless, to further evaluate the influence of an imbalanced grouping, we used median headache days of the training/test cohort (i.e., 5 headache days per month) to evenly subdivide this cohort (i.e., 77 participants of the MOl group and 74 participants of the MOh group). The results showed that the best model failed to predict migraine attack frequency in an independent sample. The median headache days were derived from the current training/test cohort rather than a larger sample, which may account for the poor classification performance in the independent dataset. We also speculated that the migraineurs who have a headache attack frequency of less than 8 days per month might share a common or similar functional connectivity pattern, resulting in the misclassification from the MOh group to the MOl group. The current results may indicate that the frequency of 8 headache days/month was more suitable to judge whether a migraineur had a lower or higher migraine attack frequency from a data‐driven analysis aspect, consistent with clinical prophylactic treatment guidelines (Silberstein et al., 2008).

### 4.3 Migraine attack frequency classification‐related features

In the present study, by using a multivariate analysis approach, discriminative functional connections were found to be related to many brain areas (i.e., the temporal lobe, limbic lobe, prefrontal lobe, and insula) that belong to different functional brain systems, rather than with activity in dedicated “pain centers” within the brain. Previous studies have repeatedly shown that multiple brain regions are involved in sensory, emotional, and evaluative aspects of pain processing in healthy individuals (Martucci & Mackey, 2018). These regions of the brain include the primary and secondary somatosensory cortices, primary and supplementary motor cortices, anterior cingulate cortex, prefrontal and parietal cortices, and limbic system (Martucci & Mackey, 2018). Additionally, abnormal brain activation of the limbic lobe, frontal lobe and temporal lobe was also observed in chronic pain, including migraine (Schwedt & Dodick, 2009). Insights from positron‐emission tomography have observed that the limbic regions were activated during migraine attacks (Afridi et al., 2005; Afridi et al., 2005; Matharu et al., 2004). It suggested that the limbic regions may modulate the affective components of pain in migraineurs, and may play an important role in the association between chronic migraine and psychiatric disturbances. Kucyi and Davis (2015) pointed out that the prefrontal cortex may be act as an “exchange hinge” for distributing pain‐related information and relate to pain‐attention interactions in chronic pain. Recently, our group found that baseline gray matter volume of the medial prefrontal cortex and its functional connectivity could predict a future placebo response in an 8‐week sham acupuncture treatment for migraine (Liu et al., 2017; Liu, Ma, et al., 2017). However, there is little specificity or congruence of these regions across studies in migraine processing.

Looking at the brain as an integrative complex system, these discriminative features were optimized to jointly predict the patients' status, suggesting that migraine attacks may be associated with an abnormal integrated network configuration, rather than in one or more isolated brain circuits. It highlighted the importance of data‐driven analysis methods, which do not restrain the features to a priori nodes of interest. Our findings were built upon previous literature suggesting that multivariate analysis approaches in classifying functional connectivity data offered a valuable technique in understanding network‐level differences in migraine attack frequency‐related neurobiology and extended previous findings by demonstrating the heterogeneity in resting‐state networks of individuals with migraine with different headache frequency (Vallières et al., 2015; Zhou et al., 2017). Our results also demonstrated good utility for distinguishing different patient groups, which may have potential to further understand the functional mechanisms contributing to the development and maintenance of migraine.

It should be noted that our results only indicated that the observed network features could identify the lower and higher migraine attacks migraineurs. It was not necessarily implied that the neural activity from the limbic lobe, frontal lobe and temporal lobe was derived corresponding to the neural activity generating migraine. As our current neuroimaging investigation is not a longitudinal study, it cannot be determined whether the observed functional connection pattern is preexisting to the continual migraine attacks, or whether the continual migraine attacks result in the abnormal functional connection pattern within the brain. Nonetheless, these discriminative features were tested on different data sets (i.e., the training/test cohort and validating cohort) by combining the bootstrap and permutation methods. The results demonstrated that brain functional features were robust in classifying the MOl group and MOh group, and random features could not successfully discriminate the MOl group from the MOh group. Finally, further investigations would provide an opportunity to corroborate and extend our findings by using additional cohorts and other neuroimaging features.

## CONFLICT OF INTEREST

The authors declare no potential conflict of interest.

## DATA AVAILABILITY STATEMENT

The data and code that support the results of this study are available from the corresponding author upon request.

## Supporting information


**Table S1** Demographic and headache information for training/test cohort with a balanced grouping.

**Table S2** Prediction performance of the best model.

## References

[hbm24854-bib-0001] Afridi, S. , Giffin, N. , Kaube, H. , Friston, K. , Ward, N. , Frackowiak, R. , & Goadsby, P. (2005). A positron emission tomographic study in spontaneous migraine. Archives of Neurology, 62, 1270–1275. 16087768 10.1001/archneur.62.8.1270

[hbm24854-bib-0002] Afridi, S.K. , Matharu, M.S. , Lee, L. , Kaube, H. , Friston, K.J. , Frackowiak, R.S.J. , Goadsby, P.J. (2005) A PET study exploring the laterality of brainstem activation in migraine using glyceryl trinitrate. Brain, 128:932–939. 15705611 10.1093/brain/awh416

[hbm24854-bib-0003] Berger, S. E. , Vachon‐Presseau, É. , Abdullah, T. B. , Baria, A. T. , Schnitzer, T. J. , & Apkarian, A. V. (2018). Hippocampal morphology mediates biased memories of chronic pain. NeuroImage, 166, 86–98. 29080714 10.1016/j.neuroimage.2017.10.030 PMC5813825

[hbm24854-bib-0004] Bernardy, K. , Lehmann, K. , Einsle, F. , Goßrau, G. , Michel, S. , & Köllner, V. (2007). Prädiktoren der Anfallshäufigkeit bei Patienten mit Migräne. Psychotherapie·Psychosomatik·Medizinische Psychologie, 57, 281–288. 10.1055/s-2006-951951 17334974

[hbm24854-bib-0005] Borsook, D. , Maleki, N. , Becerra, L. , & McEwen, B. (2012). Understanding migraine through the lens of maladaptive stress responses: A model disease of allostatic load. Neuron, 73, 219–234. 22284178 10.1016/j.neuron.2012.01.001

[hbm24854-bib-0006] Dodick, D. W. (2018). Migraine. Lancet, 391, 1315–1330. 29523342 10.1016/S0140-6736(18)30478-1

[hbm24854-bib-0007] Fan, L. , Li, H. , Zhuo, J. , Zhang, Y. , Wang, J. , Chen, L. , … Laird, A. R. (2016). The human brainnetome atlas: A new brain atlas based on connectional architecture. Cerebral Cortex, 26, 3508–3526. 27230218 10.1093/cercor/bhw157 PMC4961028

[hbm24854-bib-0008] Fox, M. D. , & Raichle, M. E. (2007). Spontaneous fluctuations in brain activity observed with functional magnetic resonance imaging. Nature Reviews. Neuroscience, 8, 700–711. 17704812 10.1038/nrn2201

[hbm24854-bib-0009] Haywood, K. L. , Mars, T. S. , Potter, R. , Patel, S. , Matharu, M. , & Underwood, M. (2018). Assessing the impact of headaches and the outcomes of treatment: A systematic review of patient‐reported outcome measures (PROMs). Cephalalgia, 38, 1374–1386. 28920448 10.1177/0333102417731348 PMC6024352

[hbm24854-bib-0010] Houle, T. T. , Turner, D. P. , Houle, T. A. , Smitherman, T. A. , Martin, V. , Penzien, D. B. , & Lipton, R. B. (2013). Rounding behavior in the reporting of headache frequency complicates headache chronification research. Headache, 53, 908–919. 23721238 10.1111/head.12126 PMC4546843

[hbm24854-bib-0011] Huang, Y.‐q. , Liang, C.‐h. , He, L. , Tian, J. , Liang, C.‐s. , Chen, X. , … Liu, Z.‐y. (2016). Development and validation of a radiomics nomogram for preoperative prediction of lymph node metastasis in colorectal cancer. Journal of Clinical Oncology, 34, 2157–2164. 27138577 10.1200/JCO.2015.65.9128

[hbm24854-bib-0012] Kucyi, A. , & Davis, K. D. (2015). The dynamic pain connectome. Trends in Neurosciences, 38, 86–95. 25541287 10.1016/j.tins.2014.11.006

[hbm24854-bib-0013] Lipton, R. B. , Bigal, M. E. , Diamond, M. , Freitag, F. , Reed, M. , & Stewart, W. F. (2007). Migraine prevalence, disease burden, and the need for preventive therapy. Neurology, 68, 343–349. 17261680 10.1212/01.wnl.0000252808.97649.21

[hbm24854-bib-0014] Lipton, R. B. , Fanning, K. M. , Serrano, D. , Reed, M. L. , Cady, R. , & Buse, D. C. (2015). Ineffective acute treatment of episodic migraine is associated with new‐onset chronic migraine. Neurology, 84, 688–695. 25609757 10.1212/WNL.0000000000001256 PMC4336107

[hbm24854-bib-0015] Lipton, R. B. , & Silberstein, S. D. (2015). Episodic and chronic migraine headache: Breaking down barriers to optimal treatment and prevention. Headache, 55, 103–122. 25662743 10.1111/head.12505_2

[hbm24854-bib-0016] Liu, J. , Ma, S. , Mu, J. , Chen, T. , Xu, Q. , Dun, W. , … Zhang, M. (2017). Integration of white matter network is associated with interindividual differences in psychologically mediated placebo response in migraine patients. Human Brain Mapping, 38, 5250–5259. 28731567 10.1002/hbm.23729 PMC6867162

[hbm24854-bib-0017] Liu, J. , Mu, J. , Liu, Q. , Dun, W. , Zhang, M. , & Tian, J. (2017). Brain structural properties predict psychologically mediated hypoalgesia in an 8‐week sham acupuncture treatment for migraine. Human Brain Mapping, 38, 4386–4397. 28608601 10.1002/hbm.23667 PMC6866832

[hbm24854-bib-0018] Liu, J. , Zhao, L. , Lei, F. , Zhang, Y. , Yuan, K. , Gong, Q. , … Tian, J. (2015). Disrupted resting‐state functional connectivity and its changing trend in migraine suffers. Human Brain Mapping, 36, 1892–1907. 25640857 10.1002/hbm.22744 PMC6869678

[hbm24854-bib-0019] Maleki, N. , Becerra, L. , Brawn, J. , Bigal, M. , Burstein, R. , & Borsook, D. (2012). Concurrent functional and structural cortical alterations in migraine. Cephalalgia, 32, 607–620. 22623760 10.1177/0333102412445622 PMC3846436

[hbm24854-bib-0020] Martucci, K. T. , & Mackey, S. C. (2018). Neuroimaging of PainHuman evidence and clinical relevance of central nervous system processes and modulation. Anesthesiology, 128, 1241–1254. 29494401 10.1097/ALN.0000000000002137 PMC5953782

[hbm24854-bib-0021] Matharu, M. S. , Thorsten, B. , Nick, W. , Frackowiak, R. S. J. , Richard, W. , & Goadsby, P. J. (2004). Central neuromodulation in chronic migraine patients with suboccipital stimulators: A PET study. Brain, 127, 220–230. 14607792 10.1093/brain/awh022

[hbm24854-bib-0022] Niere, K. , & Jerak, A. (2004). Measurement of headache frequency, intensity and duration: Comparison of patient report by questionnaire and headache diary. Physiotherapy Research International, 9, 149–156. 15790252 10.1002/pri.318

[hbm24854-bib-0023] Plitt, M. , Barnes, K. A. , Wallace, G. L. , Kenworthy, L. , & Martin, A. (2015). Resting‐state functional connectivity predicts longitudinal change in autistic traits and adaptive functioning in autism. Proceedings of the National Academy of Sciences of the United States of America, 112, E6699–E6706. 26627261 10.1073/pnas.1510098112 PMC4672806

[hbm24854-bib-0024] Power, J. D. , Barnes, K. A. , Snyder, A. Z. , Schlaggar, B. L. , & Petersen, S. E. (2012). Spurious but systematic correlations in functional connectivity MRI networks arise from subject motion. NeuroImage, 59, 2142–2154. 22019881 10.1016/j.neuroimage.2011.10.018 PMC3254728

[hbm24854-bib-0025] Robnik‐Šikonja, M. , & Kononenko, I. (2003). Theoretical and empirical analysis of ReliefF and RReliefF. Machine Learning, 53, 23–69.

[hbm24854-bib-0026] Rosenberg, M. D. , Finn, E. S. , Scheinost, D. , Papademetris, X. , Shen, X. , Constable, R. T. , & Chun, M. M. (2016). A neuromarker of sustained attention from whole‐brain functional connectivity. Nature Neuroscience, 19, 165–171. 26595653 10.1038/nn.4179 PMC4696892

[hbm24854-bib-0027] Schwedt, T. J. , Chiang, C.‐C. , Chong, C. D. , & Dodick, D. W. (2015). Functional MRI of migraine. Lancet Neurology, 14, 81–91. 25496899 10.1016/S1474-4422(14)70193-0 PMC11318354

[hbm24854-bib-0028] Schwedt, T. J. , & Dodick, D. W. (2009). Advanced neuroimaging of migraine. Lancet Neurology, 8, 560–568. 19446275 10.1016/S1474-4422(09)70107-3 PMC3645468

[hbm24854-bib-0029] Silberstein, S. , Tfelt‐Hansen, P. , Dodick, D. , Limmroth, V. , Lipton, R. , Pascual, J. , … Subcommittee, T. F.o.t. I. H. S. C. T. (2008). Guidelines for controlled trials of prophylactic treatment of chronic migraine in adults. Cephalalgia, 28, 484–495. 18294250 10.1111/j.1468-2982.2008.01555.x

[hbm24854-bib-0030] Turner, D. P. , Smitherman, T. A. , Penzien, D. B. , Lipton, R. B. , & Houle, T. T. (2013). Rethinking headache chronification. Headache, 53, 901–907. 23721237 10.1111/head.12127 PMC4546844

[hbm24854-bib-0031] Vallières, M. , Freeman, C. R. , Skamene, S. R. , & El Naqa, I. (2015). A radiomics model from joint FDG‐PET and MRI texture features for the prediction of lung metastases in soft‐tissue sarcomas of the extremities. Physics in Medicine and Biology, 60, 5471–5496. 26119045 10.1088/0031-9155/60/14/5471

[hbm24854-bib-0032] Wang, J. , Wang, X. , Xia, M. , Liao, X. , Evans, A. , & He, Y. (2015). GRETNA: A graph theoretical network analysis toolbox for imaging connectomics. Frontiers in Human Neuroscience, 9, 386. 26175682 10.3389/fnhum.2015.00386 PMC4485071

[hbm24854-bib-0033] Yuan, K. , Zhao, L. , Cheng, P. , Yu, D. , Zhao, L. , Dong, T. , … von Deneen, K. M. (2013). Altered structure and resting‐state functional connectivity of the basal ganglia in migraine patients without aura. The Journal of Pain, 14, 836–844. 23669074 10.1016/j.jpain.2013.02.010

[hbm24854-bib-0034] Zeng, L.‐L. , Shen, H. , Liu, L. , Wang, L. , Li, B. , Fang, P. , … Hu, D. (2012). Identifying major depression using whole‐brain functional connectivity: A multivariate pattern analysis. Brain, 135, 1498–1507. 22418737 10.1093/brain/aws059

[hbm24854-bib-0035] Zhou, H. , Vallières, M. , Bai, H. X. , Su, C. , Tang, H. , Oldridge, D. , … Tao, Y. (2017). MRI features predict survival and molecular markers in diffuse lower‐grade gliomas. Neuro‐Oncology, 19, 862–870. 28339588 10.1093/neuonc/now256 PMC5464433

