# Supplementary material for: Neuroimaging features of whole‐brain functional connectivity predict attack frequency of migraine
Source: Hum Brain Mapp. 2019 Nov 4;41(4):984–93. doi: 10.1002/hbm.24854 (PMC7267923; doi:10.1002/hbm.24854)
Supplement: Supplementary file 1 — Table S1 Demographic and headache information for training/test cohort with a balanced grouping. [file HBM-41-984-s001.doc]

| **Table S1. Demographic and headache information for training/test cohort with a balanced grouping** | | | |
| --- | --- | --- | --- |
|  | MOl (N=77) | MOh (N=74) | *p* |
| Age (years) | 28.16 ± 1.08 | 29.24 ± 1.27 | 0.51§ |
| Sex (M/F) | 19/58 | 20/54 | 0.74† |
| Education (years) | 15.21 ± 0.19 | 15.32 ± 0.25 | 0.90§ |
| Disease duration (monthes) | 95.10 ± 8.22 | 98.19 ± 8.71 | 0.80§ |
| Migraine attacks during past four weeks | | | |
| Headache days | 2.74 ± 0.13 | 8.70 ± 0.39 | 0.00§ |
| Average duration of a migraine attack (hours) | 10.70 ± 1.41 | 9.61 ± 0.99 | 0.53§ |
| Average pain intensity (0–10) | 5.43 ± 0.19 | 5.56 ± 0.19 | 0.64§ |
| SAS | 44.52 ± 1.05 | 47.96 ± 1.15 | 0.03§ |
| SDS | 42.91 ± 1.25 | 46.48 ± 1.27 | 0.05§ |
| MOl, migraineurs without aura with a lower attack frequency (i.e. headache days **< 5**); MOh, migraineurs without aura with a higher attack frequency (i.e. headache days **≥ 5**); SAS, self-rating anxiety scale; SDS, self-rating depression scale. § *P*-value established through a two sample *t*-test. † *P*-value established through a Chi square test. Data are presented as mean ± SE. | | | |
