# Supplementary material for: Neuroimaging features of whole‐brain functional connectivity predict attack frequency of migraine
Source: Hum Brain Mapp. 2019 Nov 4;41(4):984–93. doi: 10.1002/hbm.24854 (PMC7267923; doi:10.1002/hbm.24854)
Supplement: Supplementary file 2 — Table S2 Prediction performance of the best model. [file HBM-41-984-s002.doc]

| **Table S2. Prediction performance of the best model** | | |
| --- | --- | --- |
|  | Mean | 95% CI |
| AUC | 0.81 | [0.73, 0.86] |
| Sensitivity | 73.58% | [61.60%, 83.65%] |
| Specificity | 74.47% | [62.67%, 83.93%] |
| Accuracy | 74.05% | [67.47%, 79.80%] |
| One thousand bootstrap samplings. AUC, the area under the receiver operating characteristic curve; CI, confidence interval. | | |
